# Effect of repeated *in vivo* microCT imaging on the properties of the mouse tibia

**DOI:** 10.1371/journal.pone.0225127

**Published:** 2019-11-21

**Authors:** Sara Oliviero, Mario Giorgi, Peter J. Laud, Enrico Dall’Ara

**Affiliations:** 1 Department of Oncology and Metabolism, University of Sheffield, Sheffield, United Kingdom; 2 Insigneo Institute for *in silico* Medicine, University of Sheffield, Sheffield, United Kingdom; 3 Certara QSP, Certara UK Ltd., Simcyp Division, Sheffield, United Kingdom; 4 Statistical Services Unit, University of Sheffield, Sheffield, United Kingdom; 5 MRC Arthritis Research UK Centre for Integrated research into Musculoskeletal Ageing (CIMA), University of Sheffield, Sheffield, United Kingdom; University of Notre Dame, UNITED STATES

## Abstract

In longitudinal studies, *in vivo* micro-Computed Tomography (microCT) imaging is used to investigate bone changes over time due to interventions in mice. However, ionising radiation can provoke significant variations in bone morphometric parameters. In a previous study, we evaluated the effect of reducing the integration time on the properties of the mouse tibia measured from microCT images. A scanning procedure (100 ms integration time, 256 mGy nominal radiation dose) was selected as the best compromise between image quality and radiation dose induced on the animal. In this work, the effect of repeated *in vivo* scans has been evaluated using the selected procedure. The right tibia of twelve female C57BL/6 (six wild type, WT, six ovariectomised, OVX) and twelve BALB/c (six WT, six OVX) mice was scanned every two weeks, starting at week 14 of age. At week 24, mice were sacrificed and both tibiae were scanned. Standard trabecular and cortical morphometric parameters were calculated. The spatial distribution of densitometric parameters (e.g. bone mineral content) was obtained by dividing each tibia in 40 partitions. Stiffness and strength in compression were estimated using homogeneous linear elastic microCT-based micro-Finite Element models. Differences between right (irradiated) and left (non-irradiated control) tibiae were evaluated for each parameter. The irradiated tibiae had higher Tb.Th (+3.3%) and Tb.Sp (+11.6%), and lower Tb.N (-14.2%) compared to non-irradiated tibiae, consistently across both strains and intervention groups. A reduction in Tb.BV/TV (-14.9%) was also observed in the C57BL/6 strain. In the OVX group, a small reduction was also observed in Tt.Ar (-5.0%). In conclusion, repeated microCT scans (at 256 mGy, 5 scans, every two weeks) had limited effects on the mouse tibia, compared to the expected changes induced by bone treatments. Therefore, the selected scanning protocol is acceptable for measuring the effect of bone interventions *in vivo*.

## Introduction

*In vivo* micro-Computed Tomography (microCT) imaging is considered the gold standard for preclinical investigations of bone properties in rodents [1, 2]. Longitudinal studies where the tibia is scanned at multiple time points have been applied to investigate the effect of aging [3], drug treatments [4, 5] and mechanical loading [6, 7].

However, in previous studies a significant effect of ionising radiation on trabecular or cortical morphometric parameters has been observed, both in the mouse [8–10] and in the rat [11]. A review of these effects on the mouse tibia has been reported in Oliviero et al. [12] and is briefly summarised here. Klink et al. [8] reported that ionising radiation (846 mGy radiation dose) induced changes in the mouse tibia (12 weeks old C57BL/6J, BALB/cBy and C3H/HeJ mice) after five weekly scans. They reported significant reductions in trabecular bone volume fraction (Tb.BV/TV, 8–20%) and trabecular number (Tb.N, 9–16%), and significant increases in trabecular separation (Tb.Sp, 14–20% in BALB/cBtJ mice). Similar effects were reported by Willie et al. [10], who observed significant decreases in Tb.BV/TV (20–38%) and increases in Tb.Sp (29–39%) in young C57BL/6J mice (10 weeks old) of two different groups after four scans (every five days; 55 kVp, 145 μA, 10.5 μm voxel size, 600 ms integration time, no frame averaging, radiation dose not reported). No significant effect was found in older mice (26 weeks of age). Laperre et al. [9] found that three scans (every two weeks) at 776 mGy induced significant reductions in Tb.BV/TV (-30%) and Tb.N (-35%) in C57Bl/6J mice (10 weeks of age). Radiation effects were not significant for scans performed with 434 mGy nominal radiation dose. Lastly, the study by Sacco et al. [13] reported no significant radiation effects in male and female CD-1 mice scanned *in vivo* twice (every two months) at three different doses of radiation (222, 261 and 460 mGy).

It should be noticed that in the above studies only a portion of the tibia was scanned, usually a volume of interest of approximately 1 mm [10] in the proximal tibia, and in some cases a similar volume of interest at the midshaft. However, radiation effects are expected to increase if a larger portion of the tibia is scanned. Also, a higher number of scans over time increases the potential negative effects, despite providing more accurate information about the temporal variations in the bone properties. Therefore, there is a need to evaluate the effect of radiation on the bone properties for every newly defined scanning protocol.

In a previous study [12], we have analysed the accuracy of the image-based measurements of the mouse tibia parameters using four different scanning procedures, characterized by decreasing nominal radiation dose. The results showed that a low-radiation scanning procedure (256 mGy nominal radiation dose) provided an acceptable accuracy in the evaluation of the morphometric, densitometric and mechanical properties of the mouse tibia.

The goal of this study was to evaluate the effect of repeated *in vivo* microCT scans with the developed protocol on the properties of the mouse tibia.

## Materials and methods

### Animal study

Twelve 13-weeks-old C57BL/6 (B6) and twelve BALB/c (BAL) female mice were purchased from Envigo RMS Ltd (Bicester, UK) and used in a parallel study that aimed to evaluate the effect of ovariectomy as osteoporosis animal model [14]. Prior to the experiment, the mice were allowed to acclimate to the new environment for one week, and subsequently housed in the same environmentally controlled conditions at the Biological Services Unit of the University of Sheffield with a twelve-hour light/dark cycle at 22°C, and free access to food and water. All the procedures were performed under a British Home Office project licence (PPL 40/3499) and in compliance with the UK Animals (Scientific Procedures) Act 1986. The study was reviewed and approved by the local Research Ethics Committee of the University of Sheffield (Sheffield, UK). Six mice per strain underwent the ovariectomy (OVX) surgery at the age of 14 weeks. Therefore, four groups were considered in this study: B6 mice that underwent OVX (B6-OVX), BAL mice that underwent OVX (BAL-OVX) and the respective control wild type (WT) groups (B6-WT and BAL-WT).

**Fig 1:**
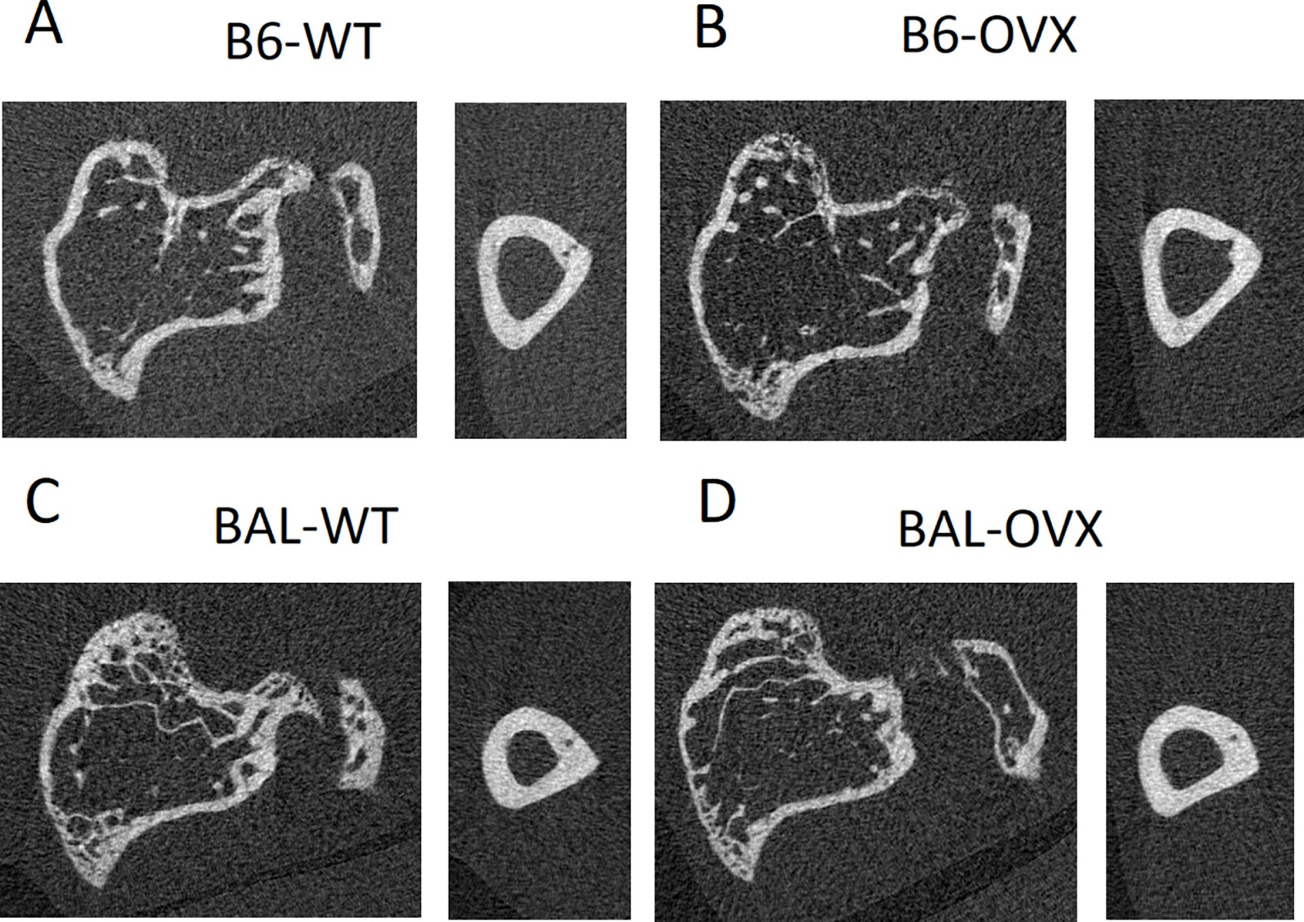
Examples of microCT cross-sections. B6 = C57BL/6 mice, BAL = BALB/c mice, WT = wild type, OVX = ovariectomy.

Details of the longitudinal study are reported in [14] and briefly summarised here. From week 14 to 22 of age, every two weeks each mouse was anaesthetised by isoflurane inhalation and the right tibia was scanned (VivaCT 80, Scanco Medical, Bruettisellen, Switzerland) *in vivo*. The scanning protocol was defined in a previous study (55 kVp, 145 μA, 10.4 μm voxel size, 100 ms integration time, 32 mm field of view, 750 projections/180°, no frame averaging, 0.5 mm Al filter) [12] as the best compromise between measurement accuracy and nominal radiation dose. In that study, the measurements obtained with different protocols have been compared to those obtained from high resolution images (4.3 μm voxel size). Based on the results, the integration time has been reduced from 200 ms (used in a previous study conducted in our laboratory [5]) to 100 ms, which results in a refined animal procedure characterised by lower radiation exposure and shorter anesthesia. The nominal radiation dose associated with this scanning protocol was 256 mGy and the total scanning time for each tibia was 25 minutes. Examples of microCT cross-sections are reported in Fig 1. The positioning of the animal in a special bed and holder allowed to irradiate only the scanned leg, while the rest of the body is shielded by a proper separative wall. At week 24 of age, mice were sacrificed by cervical dislocation while under anaesthesia. Both tibiae were scanned without dissecting the bones from the body, in a similar condition as the *in vivo* scans and with the same scanning parameters. The left tibia was used as non-irradiated control for the subsequent analyses. All images were reconstructed using the software provided by the manufacturer (Scanco Medical AG) and applying a polynomial beam hardening correction based on a phantom of 1200 mg HA/cc density, which has been shown to improve the local tissue mineralization measurement [15].

### Image processing

**Fig 2:**
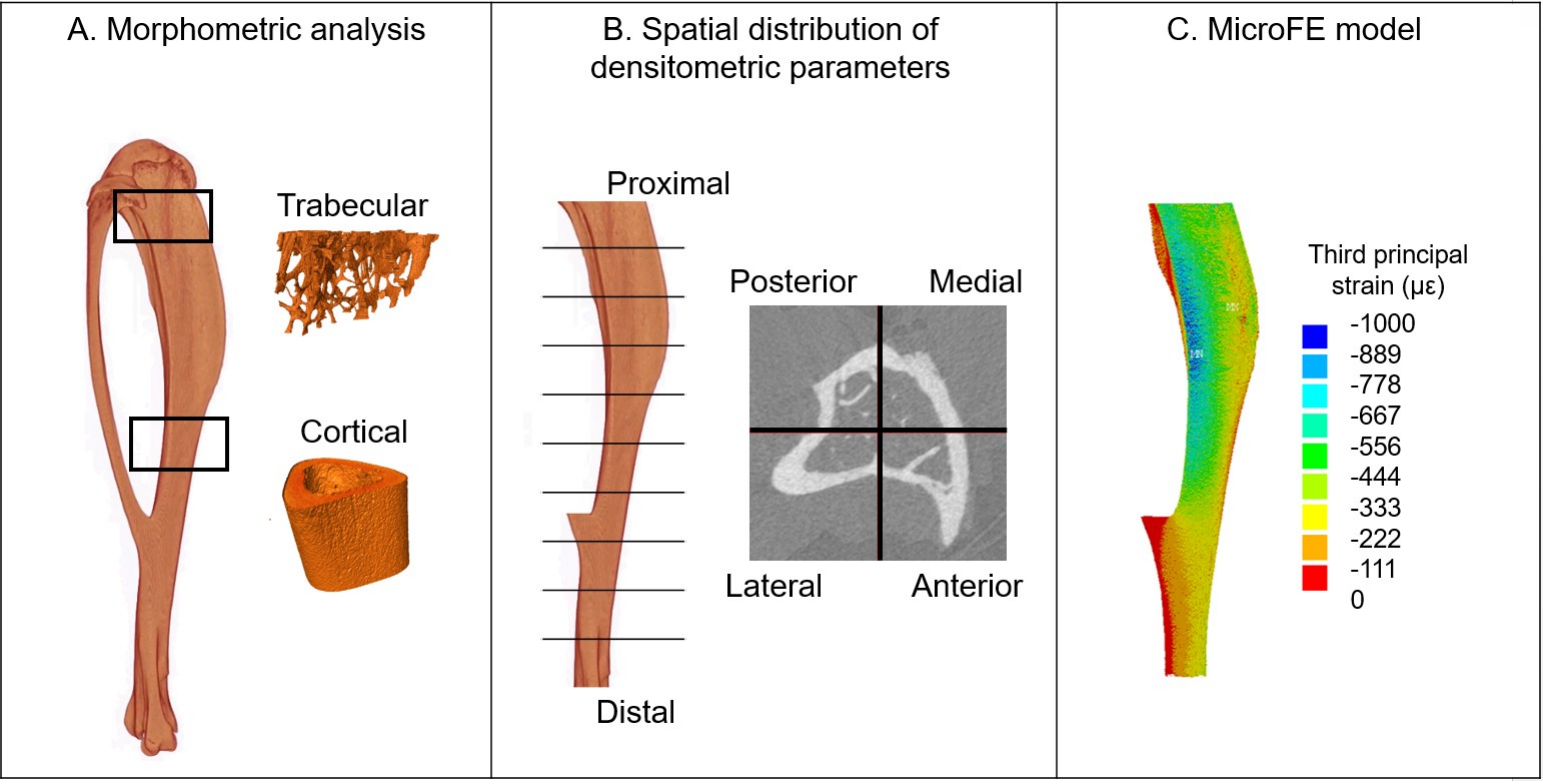
Overview of the analyses performed for each tibia. A. Standard morphometric analysis of trabecular and cortical volumes of interest. B. Spatial distribution of densitometric parameters: bone mineral content (BMC), tissue mineral density (TMD), bone mineral density (BMD), bone volume fraction (BV/TV). C. Micro-Finite Element (microFE) models for estimating the mechanical properties.

MicroCT images were used to estimate the following parameters of interest (Fig 2): standard trabecular and cortical morphometric parameters [1], spatial distribution of densitometric parameters [16] and mechanical properties in compression estimated using micro-Finite Element (microFE) models [17]. The image processing methods have been reported in a previous study [12] and are summarized here.

In order to align all images in the same reference system, a rigid registration procedure was applied. One tibia from the B6 group and one from the BAL group, scanned at week 14, were rotated in Amira (Amira 6.0.0, Thermo Fisher Scientific) in order to align their longitudinal axes to the Z-axis of a global reference system [5]. Afterwards, the images acquired at the last time point (week 24) were rigidly registered to the aligned ones. Left tibiae were horizontally flipped to perform the registration to the right ones. Normalized Mutual Information was used as optimization criterion and Lanczos interpolator was used for resampling the images [18, 19]. A Gaussian filter (kernel 3x3x3, standard deviation 0.65) was applied to reduce the high frequency noise [1].

### Standard morphometric analysis

Tibia length was computed as the distance between the most proximal and distal bone voxels in the registered images.

Morphometric analyses were performed using CTAn (v1.18.4.0, Skyscan-Bruker, Kontich, Belgium). For trabecular measurements, a volume of interest (VOI) of 1 mm was selected (Fig 2A) below the growth plate with an offset of 0.2 mm from a reference slice, identified as the point where the medial and lateral sides of the growth plate merged [16]. Trabecular bone was contoured by manually drawing 2D regions of interest every five slices. For segmentation, a single level threshold was used, calculated as the average of the grey levels corresponding to the bone and background peaks in the image histogram [20, 21]. A despeckling filter was applied to remove 3D bone volumes smaller than 10 voxels. Trabecular bone volume fraction (Tb.BV/TV), thickness (Tb.Th), separation (Tb.Sp) and number (Tb.N) were computed for each VOI [1].

For cortical analysis, a VOI of 1 mm was selected, centred at the tibial midshaft (Fig 2A). After segmentation, pores within the cortex were removed by applying a closing function (2D round kernel, radius equal to 10 pixels). Total cross-sectional area (Tt.Ar), cortical bone area (Ct.Ar), cortical area fraction (Ct.Ar/Tt.Ar) and cortical thickness (Ct.Th) were computed [1].

### Spatial distribution of bone mineral content (BMC)

The procedure for evaluating the spatial distribution of bone mineral content (BMC) over the tibia has been previously described in [12, 16]. Briefly, the registered greyscale images were converted into tissue mineral density (TMD) images by using the calibration curve provided by the manufacturer of the *in vivo* scanner. Weekly quality checks were performed on a densitometric phantom with five insertions of known equivalent density (800, 400, 200, 100 and 0 mg HA/cc) in order to check the accuracy of the calibration curve. BMC in each voxel was calculated as its TMD multiplied by the volume of the voxel.

A VOI was defined below the growth plate (Fig 2B), starting from the slice where the growth plate tissue was not visible anymore. It included the 80% of the total length of the tibia and excluded the fibula. The VOI was divided into ten longitudinal sections and four quadrants (anterior, posterior, lateral and medial). Therefore, a total of 40 partitions were obtained for each tibia. For each partition, BMC was calculated as the sum of the BMC in the voxels classified as bone [16]. Additionally, for each partition the average TMD, bone mineral density (BMD, calculated as the total BMC divided by the total volume of the partition), and bone volume fraction (BV/TV, calculated as the segmented bone volume divided by the total volume of the partition) were obtained.

### Micro-Finite Element (microFE) models

Subject-specific micro-Finite Element (microFE) models (Fig 2C) were generated from the VOI described in the previous paragraph, which was binarised using a single level threshold as described. A connectivity filter was applied in order to remove unconnected voxels (connectivity rule = 6, bwlabeln function, MATLAB R2017b, The MathWorks, Inc. USA). Hexahedral microFE models were generated by converting each bone voxel into an 8-noded hexahedral element [20]. Homogeneous isotropic linear elastic material properties (Young’s Modulus of 14.8 GPa and Poisson’s ratio of 0.3) were assigned [22].

In order to evaluate the stiffness of the bone, the proximal end of the tibia was fully constrained, while a displacement equal to 1 mm was applied on each node of the distal surface in the longitudinal direction. The apparent stiffness was calculated as the sum of reaction forces at the proximal surface, divided by the applied displacement.

For strength estimation, the proximal surface of the tibia was fully constrained, while 1 N load was applied on the distal surface, equally distributed on each node. Strength was calculated by assuming that the tibia failed when 2% of the nodes reached a critical strain level (adapted from [23]) of either -10300 με in compression or +8000 με in tension [24]. The 10% of the total length was excluded from this analysis at each extremity of the model in order to reduce the boundary effects.

The outputs of these models have been recently validated against state of the art experiments for the measurement of local displacements, using a combination of microCT scanning, *in situ* mechanical testing and digital volume correlation [17]. That study showed an optimal ability of the models to predict displacements (R^2^>0.82, RMSE< 22%), stiffness (differences equal to 14%±11%) and strength (differences equal to 9%±9%).

All microFE models were solved (Ansys, Release 15.0, ANSYS, Inc.) on an HPC system (HPC Beagle, INSIGNEO, University of Sheffield; 64 cores, memory = 128GB) in approximately 70 minutes (5 hours CPU time).

### Statistical analysis

The effect of radiation exposure was evaluated by comparing each of the bone parameters obtained for the right irradiated limb to the left non-irradiated one.

For each morphometric parameter, tibia length, Total BMC and estimated mechanical properties, a linear mixed effects ANOVA model was fitted to the pooled data (N = 20) with a random effect for mouse, to investigate interactions between factors while accounting for correlations between measurements from the same mouse. The factors analysed were: Strain (B6 vs BAL), Intervention (WT vs OVX), Radiation&Side (Left non-irradiated vs Right irradiated). All two-way interaction terms were included in this model. The assumption of normality was met for all variables (Shapiro-Wilk test, p>0.05). The assumption of constant variance between intervention groups and (separately) between strains was tested using Levene’s test. Where heteroskedasticity was identified (p<0.05 for length, Tt.Ar, Ct.Ar, Tb.BV/TV), the data were split into subgroups and separate models were fitted. Patterns of non-constant variance that were inconsistent across strains or across intervention groups were disregarded as spurious. For a consistent approach, the analysis of Total BMC was also split into separate models for assessment of the Intervention*Radiation&Side interaction (p = 0.025). Subsequently, non-significant interaction terms were removed to obtain parsimonious models for estimating the effect of Radiation&Side (with 95% confidence intervals) using contrasts of estimated marginal means. All analysis was carried out in R v3.6.0.

Analyses for the single groups were discussed as trends, given the limited sample size (N = 4–6 mice/group).

## Results

One mouse from the B6-OVX and BAL-WT groups, and two from the BAL-OVX group were euthanized due to complications prior to the end of the study and thus were not included in these analyses. The interested readers can find an example of the images used in this study at https://doi.org/10.15131/shef.data.10247816 and are welcome to contact the corresponding author for having access to the whole dataset.

### Pooled data

For each parameter, the p-values for each factor and interaction term are shown in Table 1. Significant differences between Strain and/or Intervention were observed for many of the parameters. The within-mouse effect of Radiation&Side was significant (p<0.05) for Length, Tt.Ar, Tb.BV/TV, Tb.Th, Tb.Sp and Tb.N. There was a significant interaction between Intervention and Radiation&Side factors for Tt.Ar (p = 0.012), Ct.Ar (p = 0.044) and Total BMC (p = 0.025).

**Table 1:** Results from random effects ANOVA tests (coefficient p-values) obtained by pooling data from all groups.

		Intervention	Strain	Radiation &Side	Intervention* Radiation &Side	Strain* Intervention	Strain* Radiation &Side
Length	WT	-	0.964	**0.001**	-	-	0.470
	OVX	-	0.850	0.389	-	-	0.400
Tt.Ar	WT	-	**0.010**	0.744	-	-	0.851
	OVX	-	**0.015**	**0.023**	-	-	0.596
Ct.Ar	WT	-	**0.039**	0.808	-	-	0.827
	OVX	-	**0.005**	0.085	-	-	0.707
Ct.Ar/Tt.Ar		0.133	**<0.001**	0.244	0.417	0.857	0.632
Ct.Th		0.810	**<0.001**	0.521	0.418	0.604	0.281
Tb.BV/TV	B6	**0.002**	-	**0.001**	0.236	-	-
	BAL	0.053	-	0.196	0.626	-	-
Tb.Th		**0.012**	0.466	**0.041**	0.512	0.793	0.895
Tb.Sp		**0.001**	0.140	**<0.001**	0.872	0.775	0.365
Tb.N		**0.005**	**<0.001**	**<0.001**	0.880	0.775	0.844
Total BMC	WT	-	**0.012**	0.118	-	-	0.998
	OVX	-	**0.003**	0.152	-	-	0.762
Strength		0.573	**0.016**	0.425	0.765	0.780	0.324
Stiffness		0.527	0.270	0.532	0.495	0.956	0.269

Separate models were used where heteroskedasticity was identified from the full model (Levene’s test, p<0.05). For each variable the effect of three factors is reported (Intervention: WT vs OVX; Strain: C57BL/6 vs BALB/c; Radiation&Side: left non-irradiated vs right irradiated), as well as interactions between factors (indicated by *). Significant differences or significant interactions (p<0.05) are reported in bold. B6 = C57BL/6 mice, BAL = BALB/c mice, WT = wild type, OVX = ovariectomy. Parameters reported: length; trabecular parameters: bone volume fraction (Tb.BV/TV), thickness (Tb.Th), separation (Tb.Sp), number (Tb.N); cortical parameters: total area (Tt.Ar), cortical area (Ct.Ar), area fraction (Ct.Ar/Tt.Ar), thickness (Ct.Th); total bone mineral content (BMC); mechanical properties: stiffness, strength.

The estimates of differences between right and left tibiae from the simplified models (omitting non-significant interaction terms) are shown in Table 2. These differences are also expressed as a percentage of the overall estimated mean in the left tibia, averaged across strain and intervention groups as appropriate.

**Table 2:** Differences between right and left tibiae obtained by pooling data from all groups.

		L	R	Diff (95% CI)	SD	Diff [%] (95% CI)
Length [mm]	**WT**	**17.8**	**17.7**	**-0.110 (-0.161, -0.059)**	**0.076**	**-0.61 (-0.90, -0.33)**
	OVX	18.2	18.1	-0.074 (-0.278, 0.130)	0.266	-0.41 (-1.53, 0.72)
Tt.Ar [mm^2^]	WT	0.847	0.845	-0.002 (-0.017, 0.012)	0.021	-0.3 (-2.0, 1.4)
	**OVX**	**0.917**	**0.871**	**-0.046 (-0.079, -0.012)**	**0.044**	**-5.0 (-8.7, -1.3)**
Ct.Ar [mm^2^]	WT	0.559	0.558	-0.001 (-0.007, 0.005)	0.009	-0.1 (-1.2, 1.0)
	OVX	0.589	0.567	-0.023 (-0.047, 0.001)	0.031	-3.8 (-7.9, 0.2)
Ct.Ar/Tt.Ar [%]		65.5	65.9	0.378 (-0.329, 1.085)	1.51	0.6 (-0.5, 1.7)
Ct.Th [μm]		241	240	-1.224 (-4.927, 2.479)	7.91	-0.5 (-2.0, 1.0)
Tb.BV/TV [%]	**B6**	**6.40**	**5.45**	**-0.955 (-1.433, -0.476)**	**0.71**	**-14.9 (-22.4, -7.4)**
	BAL	9.31	8.50	-0.811 (-2.003, 0.381)	1.55	-8.7 (-21.5, 4.1)
Tb.Th [μm]		**53.7**	**55.4**	**1.765 (0.219, 3.312)**	**3.30**	**3.3 (0.4, 6.2)**
Tb.Sp [μm]		**358**	**400**	**41.737 (23.383, 60.092)**	**39.2**	**11.6 (6.5, 16.8)**
Tb.N [1/mm]		**1.43**	**1.23**	**-0.203 (-0.290, -0.116)**	**0.185**	**-14.2 (-20.3, -8.1)**
Total BMC [mg]	WT	11.0	11.2	0.185 (-0.040, 0.410)	0.335	1.7 (-0.4, 3.7)
	OVX	11.7	11.3	-0.364 (-0.843, 0.115)	0.622	-3.1 (-7.2, 1.0)
Strength [N]		14.2	14.7	0.489 (-0.953, 1.931)	3.08	3.4 (-6.7, 13.6)
Stiffness [N/mm]		167	171	3.836 (-13.557, 21.230)	37.2	2.3 (-8.1, 12.7)

Estimated marginal means from random effects ANOVA models for parameters measured in left (L, non-irradiated) and right (R, irradiated) tibiae, with estimated effect of Radiation&Side. Where the full model indicated a significant interaction, or a non-constant variance between groups, separate ANOVA models were used. B6 = C57BL/6 mice, BAL = BALB/c mice, WT = wild type, OVX = ovariectomy. Parameters reported: length; trabecular parameters: bone volume fraction (Tb.BV/TV), thickness (Tb.Th), separation (Tb.Sp), number (Tb.N); cortical parameters: total area (Tt.Ar), cortical area (Ct.Ar), area fraction (Ct.Ar/Tt.Ar), thickness (Ct.Th); total bone mineral content (BMC); mechanical properties: stiffness, strength.

From the above analyses, irradiated tibiae were observed to have significantly higher Tb.Th (mean difference +3.3%) and Tb.Sp (+11.6%), and lower Tb.N (-14.2%) compared to non-irradiated tibiae in the same mice, consistently across both strains and intervention groups. A reduction in Tb.BV/TV (-14.9%) was also observed in the C57BL/6 strain. In the OVX group, lower Tt.Ar (-5.0%) was observed for irradiated tibiae. A small significant reduction in the irradiated tibia length (-0.61%) was seen in WT mice. The significant Intervention*Radiation&Side interaction observed for Total BMC reflected small Radiation&Side effects that were in opposite directions for the two intervention groups, but not significantly different from zero in either group.

### Trends in standard morphometric parameters among groups

**Fig 3:**
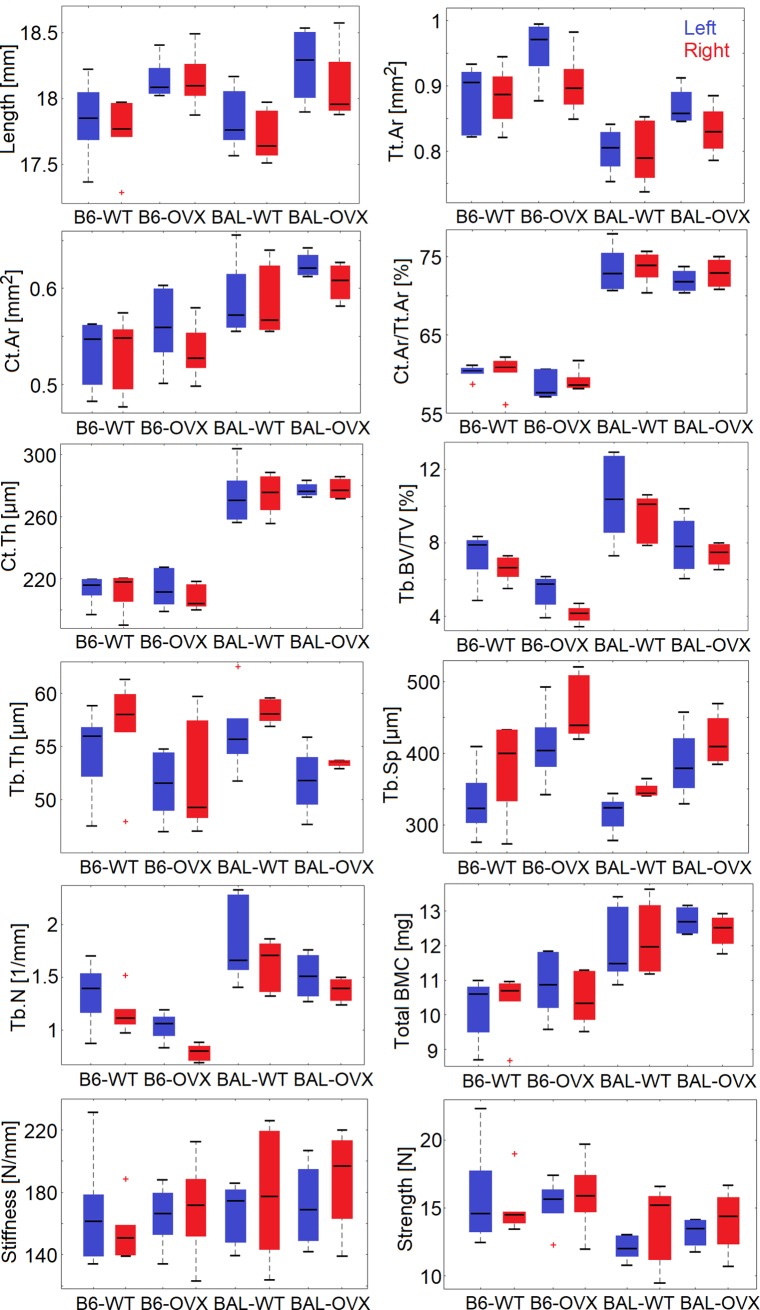
**Bone properties of irradiated (red) right tibia and non-irradiated (blue) left tibia.** B6 = C57BL/6 mice, BAL = BALB/c mice, WT = wild type, OVX = ovariectomy. Parameters reported: length; trabecular parameters: bone volume fraction (Tb.BV/TV), thickness (Tb.Th), separation (Tb.Sp), number (Tb.N); cortical parameters: total area (Tt.Ar), cortical area (Ct.Ar), area fraction (Ct.Ar/Tt.Ar), thickness (Ct.Th); total bone mineral content (BMC); mechanical properties: stiffness, strength. The median of the distribution is reported in a black line for each box, while the edges of the box represent the 25^th^ and 75^th^ percentiles. Black dashed lines extend to the most extreme values not considered as outliers. Outliers are reported in red crosses.

In Table 3 and Fig 3, comparisons between the irradiated and non-irradiated tibiae are reported for each of the four groups analysed. Some similar patterns were observed among the analysed groups. Tb.BV/TV tended to be lower in the irradiated limb. Variations observed were larger for the B6-WT (-10%±11%), the B6-OVX (-24%±8%) and the BAL-WT (-16%±18%) groups compared to the BAL-OVX (-3%±12%) group. Similarly, trabecular separation (Tb.Sp) in the irradiated tibiae tended to be higher in all groups (+11%±14% for B6-WT, +6%±8% for BAL-WT, +9%±11% for B6-OVX and +10%±14% for BAL-OVX), which corresponded to median variations of 21–35 μm. Trabecular number (Tb.N) tended to be lower (-15%±12% for B6-WT, -17%±15% for BAL-WT, -24%±10% for B6-OVX and -6%±7% for BAL-OVX) in all groups. Small variations were observed in cortical parameters for the WT groups (less than 5% for all parameters). Total cortical area (Tt.Ar) showed to be slightly lower for the OVX groups (-6%±5% for the B6-OVX and -5%±5% for the BAL-OVX) in the irradiated limb compared to the non-irradiated one, as well as cortical area (Ct.Ar) (-4%±6% for B6-OVX and -3%±4% for BAL-OVX).

**Table 3:** Trends in differences between right and left tibiae for each group.

Group		Length [mm]	Tt.Ar [mm^2^]	Ct.Ar [mm^2^]	Ct.Ar/ Tt.Ar [%]	Ct.Th [μm]	Tb. BV/TV [%]	Tb.Th [μm]	Tb.Sp [μm]	Tb.N [1/mm]	BMC [mg]	Stiffness [N/mm]	Strength [N]
B6- WT (N = 6)	L	17.9 ± 0.3	0.90 ± 0.05	0.55 ± 0.03	60 ± 1	216 ± 9	7.9 ± 1.3	56 ± 4	323 ± 47	1.39 ± 0.29	10.61 ± 0.90	161 ± 36	15 ± 4
	R	17.8 ± 0.3	0.89 ± 0.04	0.55 ± 0.04	61 ± 2	218 ± 12	6.6 ± 0.7	58 ± 5	400 ± 65	1.12 ± 0.19	10.70 ± 0.87	151 ± 18	15 ± 2
	Diff [%]	-0.5±0.5	-1 ± 2	-1 ± 1	0 ± 3	0 ± 2	-10 ± 11	2 ± 5	11 ± 14	-15 ±12	1 ± 4	-8 ± 23	0 ± 28
B6- OVX (N = 5)	L	18.1 ± 0.2	0.97 ± 0.05	0.56 ± 0.04	58 ± 2	212 ± 13	5.8 ± 0.9	52 ± 3	404 ± 54	1.06 ± 0.13	10.88 ± 0.95	166 ± 20	16 ± 2
	R	18.1 ± 0.2	0.90 ± 0.05	0.53 ± 0.03	59 ± 1	204 ± 8	4.2 ± 0.5	49 ± 6	439 ± 46	0.80 ± 0.08	10.34 ± 0.78	172 ± 32	16 ± 3
	Diff [%]	-0.5±1.5	-6 ± 5	-4 ± 6	2 ± 3	-1 ± 5	-24 ± 8	5 ± 9	9 ± 11	-24 ± 10	-1 ± 7	2 ± 15	0 ± 14
BAL- WT (N = 5)	L	17.8 ± 0.2	0.80 ± 0.03	0.57 ± 0.04	73 ± 3	271 ± 19	10.4 ± 2.4	56 ± 4	324 ± 25	1.66 ± 0.41	11.48 ± 1.11	175 ± 20	12 ± 1
	R	17.6 ± 0.2	0.79 ± 0.05	0.57 ± 0.04	74 ± 2	276 ± 13	10.1 ± 1.3	58 ± 1	344 ± 10	1.71 ± 0.25	11.97 ± 1.08	177 ± 43	15 ± 3
	Diff [%]	-0.8±0.3	0 ± 3	-1 ± 2	0 ± 3	2 ± 4	-16 ± 18	4 ± 6	6 ± 8	-17 ± 15	2 ± 3	8 ± 29	18 ± 21
BAL- OVX (N = 4)	L	18.3 ± 0.3	0.86 ± 0.03	0.62 ± 0.01	72 ± 1	276 ± 5	7.8 ± 1.6	52 ± 3	379 ± 53	1.51 ± 0.23	12.69 ± 0.42	169 ± 29	13 ± 1
	R	18.0 ± 0.3	0.83 ± 0.04	0.61 ± 0.02	73 ± 2	277 ± 7	7.5 ± 0.6	54 ± 0	410 ± 38	1.40 ± 0.11	12.52 ± 0.50	197 ± 35	14 ± 2
	Diff [%]	-0.6±1.4	-5 ± 5	-3 ± 4	2 ± 1	0 ± 1	-3 ± 12	3 ± 6	10 ± 14	-6 ± 7	-2 ± 2	2 ± 20	7 ± 13

Trends in differences (median ± SD) between right (R, irradiated) and left (L, non-irradiated) tibiae. B6 = C57BL/6 mice, BAL = BALB/c mice, WT = wild type, OVX = ovariectomy. Parameters reported: length; trabecular parameters: bone volume fraction (Tb.BV/TV), thickness (Tb.Th), separation (Tb.Sp), number (Tb.N); cortical parameters: total cross-sectional area (Tt.Ar), cortical area (Ct.Ar), area fraction (Ct.Ar/Tt.Ar), thickness (Ct.Th); total bone mineral content (BMC); mechanical properties: stiffness, strength.

### Trends in spatial distribution of bone mineral content (BMC) among groups

Median differences in total BMC were between -2% and +2% across the groups (Table 3). Percentage variations in local BMC in the 40 partitions are reported in Table 4. For three of the groups, the BMC in the lateral partitions was lower in the irradiated tibiae compared to the non-irradiated ones, with larger differences in the proximal part. No other systematic patterns were observed among the different groups for the other partitions. The largest variations were not located in the same region among different groups of mice. In B6-WT mice, the largest variation was observed in the medial partition close to the midshaft (M-04, +9%±14%). In the B6-OVX group, a difference of -13%±11% was observed in the lateral sector towards the proximal end (L-02). In BAL-WT mice, the largest variation was observed in the lateral partition close to the midshaft (L-04, -15%±11%). Lastly, in BAL-OVX mice, the largest difference was located in the most proximal lateral partition (L-01, -20%±11%).

**Table 4:** Trends in differences in local BMC between right and left tibiae for each group.

**B6-WT**					**B6-OVX**				
	**L**	**A**	**M**	**P**		**L**	**A**	**M**	**P**
**01**	1±15	3±16	0±33	3±5	**01**	-4±10	3±15	-1±19	-9±19
**02**	-3±13	1±12	1±18	5±9	**02**	-13±11	-3±10	4±10	-5±15
**03**	1±13	2±9	5±19	0±6	**03**	-7±11	-3±10	6±14	3±11
**04**	2±13	3±8	9±14	-1±6	**04**	-2±9	-8±10	4±9	-2±9
**05**	1±11	-3±8	1±6	3±12	**05**	-2±7	-2±9	-1±7	3±17
**06**	-3±9	2±5	-1±3	1±8	**06**	-3±9	2±8	-8±7	0±10
**07**	-5±12	3±2	-4±8	0±6	**07**	-6±5	0±8	-9±11	1±14
**08**	-2±7	2±3	-2±5	-3±7	**08**	-3±7	3±10	-12±10	-1±13
**09**	-3±10	0±6	-1±3	1±5	**09**	-2±7	-1±5	-10±6	-2±8
**10**	2±13	2±4	2±7	2±13	**10**	1±15	-2±7	-4±11	4±9
**BAL-WT**					**BAL-OVX**				
	**L**	**A**	**M**	**P**		**L**	**A**	**M**	**P**
**01**	-8±10	5±7	14±17	6±14	**01**	-20±11	1±8	-11±16	1±9
**02**	-5±11	5±4	8±19	7±9	**02**	-17±20	3±6	-10±9	6±9
**03**	-9±11	6±3	5±4	3±5	**03**	-10±22	1±3	3±6	4±7
**04**	-15±11	10±5	9±7	5±5	**04**	-17±17	3±4	2±4	6±6
**05**	-6±8	6±5	2±5	3±2	**05**	-17±12	3±6	-1±4	4±6
**06**	-6±8	5±3	-1±4	3±2	**06**	-12±7	4±5	-7±1	5±6
**07**	-5±8	1±3	-4±7	3±5	**07**	-9±5	-1±6	-14±5	-6±10
**08**	-3±6	4±3	-6±6	6±6	**08**	-7±3	8±3	-7±4	13±5
**09**	1±7	6±6	4±5	6±5	**09**	-3±3	7±4	-5±5	7±3
**10**	1±10	5±7	4±7	9±5	**10**	-11±7	7±12	0±11	14±6

Trends in differences in local BMC between right (irradiated) and left (non-irradiated) tibiae. B6 = C57BL/6 mice, BAL = BALB/c mice, WT = wild type, OVX = ovariectomy. Percentage differences (median ± SD) are reported for the ten longitudinal sections (01 = most proximal, 10 = most distal) and four quadrants (L = lateral, A = anterior, M = medial, P = posterior).

Percentage variations in TMD, BMD and BV/TV in the 40 partitions are reported in the supporting information (S1–S3 Tables). No consistent trends were observed among the groups.

### Trends in mechanical properties among groups

**Fig 4:**
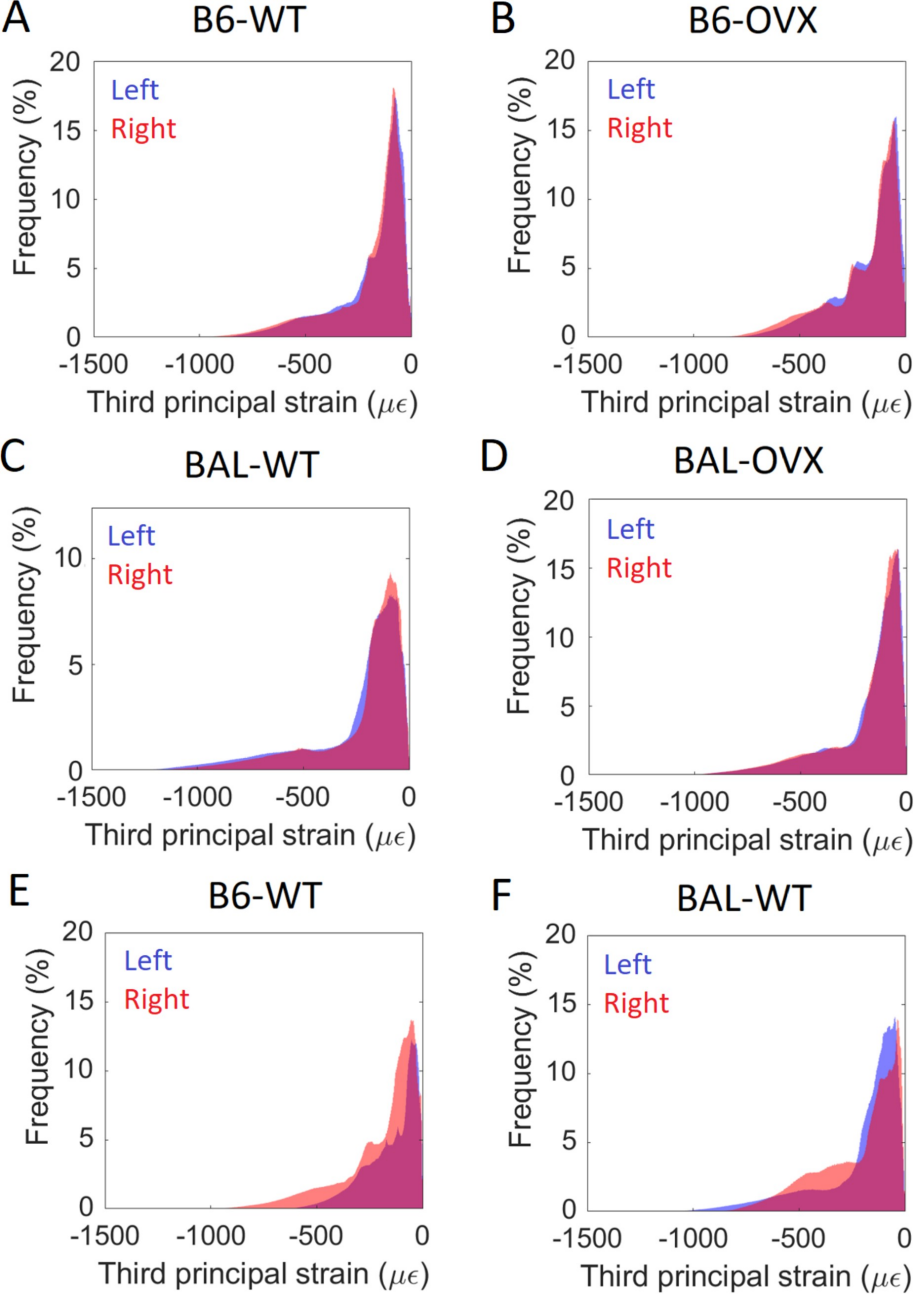
Histograms of third principal strain. B6 = C57BL/6 mice, BAL = BALB/c mice, WT = wild type, OVX = ovariectomy. In A-D, a representative mouse is reported for each of the four analysed groups. In E-F mice which showed the largest differences in local strains between the right and left tibia are reported.

Stiffness was positively but weakly correlated to total BMC (p = 0.001, R = 0.49). No correlation was found between strength and total BMC (p = 0.89), highlighting the benefit of FE modelling for evaluating the mechanical competence of the bone. Differences in global stiffness between irradiated and non-irradiated bones estimated from microFE models were: -8%±23% for B6-WT mice, 2%±15% for B6-OVX, +8%±29% for BAL-WT and +2%±20% for BAL-OVX mice. For strength, differences of 0%±28% in the B6-WT group, 0%±14% for B6-OVX, +18%±21% for BAL-WT and +7%±13% for the BAL-OVX group were observed. Strain distributions were in most cases consistent between the right and left tibiae (Fig 4A–4D), with peaks corresponding to similar strain levels. In some cases, higher peak strains were observed in the left or in the right tibia compared to the contralateral one (Fig 4E and 4F). However, no consistent increase or decrease in local strains was observed in the irradiated tibia compared to the non-irradiated one.

## Discussion

In this study the effect of repeated *in vivo* microCT scans on the properties of the mouse tibia has been evaluated for two strains (C57BL/6 and BALB/c) and two groups of mice (wild type, WT, and ovariectomised, OVX). For each mouse, the right tibia was scanned *in vivo* five times every two weeks, while the left tibia was used as non-irradiated control. The effect of radiation was evaluated on several properties of the tibia, measured from microCT images.

The irradiated tibiae had higher Tb.Th (+3.3%) and Tb.Sp (+11.6%), and lower Tb.N (-14.2%) compared to non-irradiated ones. In C57BL/6 mice, a reduction in Tb.BV/TV (-14.9%) was also observed, and in OVX mice irradiated tibiae showed a reduced Tt.Ar (-5.0%). Differences in local BMC were higher at the proximal extremity of the tibia, probably due to the observed effects in the trabecular bone for the irradiated tibia. Interestingly, for three of the groups BMC in the lateral partitions tended to be reduced in the irradiated tibia. No consistent patterns were observed for the other partitions among the different groups. Mechanical properties did not show a systematic trend in the irradiated tibia compared to the non-irradiated one. Some interaction between radiation and OVX surgery was observed, even though it was limited to three of the parameters (Tt.Ar, Ct.Ar and Total BMC), indicating that radiation effects are not strongly influenced by the metabolic state of the bone. This finding is nevertheless in contrast with a previous study [8], where no interactions between OVX surgery and radiation was observed. This difference could be due to the animals used in the studies (different age and some differences in the strains) and in the different statistical test used to identify the interactions. These findings suggest that the scanning procedure used in this study has limited impact on the bone properties even after five scans of the whole tibia, making it applicable for measuring the effect of interventions on bone remodeling. Nevertheless, a control group of animals that did not undergo any interventions is still required to avoid possible misinterpretation of the effects of the intervention alone.

In the context of the 3Rs (reduction, refinement and replacement of the usage of animals in research) with this novel procedure, the mice are subjected to a shorter scan, lower radiation exposure and shorter anesthesia (refinement of the *in vivo* microCT scanning procedure) compared to previous study [5]. This procedure has shown acceptable effects on the bone properties, while acquiring comprehensive information about the whole tibia at five different time points.

The observed effects are in line with the literature, even though methodological differences exist among different studies. Results from all studies [8–10, 13] suggest that radiation above 460 mGy/scan induces bone loss in the trabecular compartment, which seems to occur through loss of the thinner trabeculae, resulting in decreased Tb.BV/TV, increased Tb.Sp and similar or moderately increased Tb.Th. Laperre et al. [9] suggested that their scanning protocol (434 mGy/scan, 3 scans, every two weeks) is likely close to the limit of safely using for *in vivo* imaging, as consistent reductions in the trabecular bone mass were observed in the irradiated limb. Similarly, Sacco et al. [13] reported non-significant reductions in bone volume fraction (19–28%, calculated from Fig 3 [13]) and trabecular number (17–25%, calculated from Fig 3 [13]) for different protocols (222–460 mGy/scan, 2 scans, every two months). In this study, a similar or lower radiation dose (256 mGy/scan) but more scans (five, every two weeks) resulted in similar effects of radiation. Lastly, the size of the scanned volume determines the total scanning time, therefore scanning the whole tibia required an adaptation of the protocol, compared to those used for scanning a portion of the bone only. In general, each procedure needs to be tested in order to ensure that radiation effects are acceptable according to the final application. The scanning protocol defined in this study allows to scan the whole tibia at five time points with limited radiation effects, thus providing comprehensive information about bone changes in both space and time.

Limited data is reported in literature regarding the effect of radiation on densitometric and mechanical properties of the mouse tibia. In [3], no significant effects of radiation were observed on trabecular bone mineral density and tissue mineral density (188 mGy/scan, 12 scans over 42 weeks). Differences in mechanical properties observed in this study between the right and left limb were small for most mice, which is consistent with the small effects found on cortical parameters, since the overall mechanical properties of the mouse tibia under uniaxial compression are mainly determined by cortical bone [12]. For some mice, large differences were observed between the left and right tibiae (up to 42%), which however were not associated with radiation since reduced or increased mechanical properties were not consistently found for the right limb. In this study, it was assumed that the right and left tibiae had the same homogeneous material properties, therefore the observed differences were more likely associated to geometrical factors, e.g. the alignment and curvature of the tibia.

The main limitation of this study is the small sample size (N = 4–6 mice/group), which can reduce the ability of identifying significant effects of radiation in each group. Therefore, we have performed a full statistical analysis for the pooled data only (N = 20), which met the appropriate sample size. Moreover, the paired design of the experiment allowed us to increase the statistical power of the analyses to detect differences in the within-mouse comparison (effect of radiation and side). Variations in the single groups were discussed as trends only. Nevertheless, in previous studies significant differences were identified for similar sample sizes (N = 4–8 mice/group) [8–10], which corroborates the finding that the scanning protocol selected in this study had acceptable radiation effects. Another limitation is that the observed variations between the right and left limb could potentially be due to other factors in addition to radiation. The results presented in this study are based on the assumption that contralateral limbs are not significantly different at baseline, in line with previous studies for mice of different strains [25, 26]. Nevertheless, the differences observed in this study have been presented as due to both radiation and potential lateralization (Radiation&Side).

In the framework of using microCT for *in vivo* longitudinal imaging, the aim of this study was to quantify if radiation could potentially affect the longitudinal measurements of bone changes due to interventions. Higher impact on bone properties has been reported for possible interventions of interest, especially on trabecular parameters, which were more influenced by radiation. For example, *in vivo* compressive loading provoked an increase of +21–107% in trabecular bone volume, +31–68% in trabecular thickness and +13–72% in cortical cross-sectional properties [27]. Therefore, the observed radiation effects would not impair the ability to measure the bone changes of interest longitudinally. Nevertheless, it is important to have control groups in longitudinal studies, in order to account not only for the effect of growth, but also for potential effects of radiation.

In conclusion, in this study the effect of repeated *in vivo* microCT imaging has been evaluated on the mouse tibia of C57BL/6 and BALB/c female mice between 14 and 24 weeks of age. The selected scanning regime (at 256 mGy, 5 scans, every two weeks) showed limited effects on the morphometric, densitometric and mechanical properties of the mouse tibia, therefore it can be considered an adequate compromise between image quality and radiation exposure. In future studies, it will be applied for the longitudinal investigation of the effects of bone interventions, including anabolic drugs and mechanical loading, on the whole mouse tibia.

## Supporting information

S1 Table Differences in local TMD between right (irradiated) and left (non-irradiated) tibiae. (DOCX)

S2 Table Differences in local BMD between right (irradiated) and left (non-irradiated) tibiae. (DOCX)

S3 Table Differences in local BV/TV between right (irradiated) and left (non-irradiated) tibiae. (DOCX)

S1 Appendix Nominal radiation dose. (DOCX)

## References

[1] Bouxsein ML, Boyd SK, Christiansen BA, Guldberg RE, Jepsen KJ, Müller R. Guidelines for assessment of bone microstructure in rodents using micro–computed tomography. Journal of Bone and Mineral Research. 2010;25(7):1468–86. 10.1002/jbmr.141 20533309

[2] Dall’Ara E, Boudiffa M, Taylor C, Schug D, Fiegle E, Kennerley AJ, et al Longitudinal imaging of the ageing mouse. Mechanisms of Ageing and Development. 2016;160:93–116. 10.1016/j.mad.2016.08.001 27530773

[3] Buie HR, Moore CP, Boyd SK. Postpubertal architectural developmental patterns differ between the L3 vertebra and proximal tibia in three inbred strains of mice. Journal of Bone and Mineral Research. 2008;23(12):2048–59. 10.1359/jbmr.080808 18684086

[4] Campbell G, Tiwari S, Grundmann F, Purcz N, Schem C, Glüer C-C. Three-dimensional Image Registration Improves the Long-term Precision of In Vivo Micro-Computed Tomographic Measurements in Anabolic and Catabolic Mouse Models. Calcified Tissue International. 2014;94(3):282–92. 10.1007/s00223-013-9809-4 24170302

[5] Lu Y, Boudiffa M, Dall’Ara E, Liu Y, Bellantuono I, Viceconti M. Longitudinal effects of Parathyroid Hormone treatment on morphological, densitometric and mechanical properties of mouse tibia. Journal of the Mechanical Behavior of Biomedical Materials. 2017;75(Supplement C):244–51. 10.1016/j.jmbbm.2017.07.034. 28756285

[6] Birkhold AI, Razi H, Duda GN, Weinkamer R, Checa S, Willie BM. The Periosteal Bone Surface is Less Mechano-Responsive than the Endocortical. Scientific Reports. 2016;6:23480 10.1038/srep23480 https://www.nature.com/articles/srep23480#supplementary-information. 27004741 PMC4804282

[7] Holguin N, Brodt MD, Sanchez ME, Silva MJ. Aging diminishes lamellar and woven bone formation induced by tibial compression in adult C57BL/6. Bone. 2014;65:83–91. 10.1016/j.bone.2014.05.006 24836737 PMC4091978

[8] Klinck RJ, Campbell GM, Boyd SK. Radiation effects on bone architecture in mice and rats resulting from in vivo micro-computed tomography scanning. Medical Engineering & Physics. 2008;30(7):888–95. 10.1016/j.medengphy.2007.11.004 18249025

[9] Laperre K, Depypere M, van Gastel N, Torrekens S, Moermans K, Bogaerts R, et al Development of micro-CT protocols for in vivo follow-up of mouse bone architecture without major radiation side effects. Bone. 2011;49(4):613–22. 10.1016/j.bone.2011.06.031 21763477

[10] Willie BM, Birkhold AI, Razi H, Thiele T, Aido M, Kruck B, et al Diminished response to in vivo mechanical loading in trabecular and not cortical bone in adulthood of female C57Bl/6 mice coincides with a reduction in deformation to load. Bone. 2013;55(2):335–46. 10.1016/j.bone.2013.04.023 23643681

[11] Mustafy T, Benoit A, Londono I, Moldovan F, Villemure I. Can repeated in vivo micro-CT irradiation during adolescence alter bone microstructure, histomorphometry and longitudinal growth in a rodent model? PloS one. 2018;13(11):e0207323–e. 10.1371/journal.pone.0207323 . 30439999 PMC6237372

[12] Oliviero S, Lu Y, Viceconti M, Dall'Ara E. Effect of integration time on the morphometric, densitometric and mechanical properties of the mouse tibia. Journal of Biomechanics. 2017;65(Supplement C):203–11. 10.1016/j.jbiomech.2017.10.026. 29126603

[13] Sacco SM, Saint C, Longo AB, Wakefield CB, Salmon PL, LeBlanc PJ, et al Repeated irradiation from micro-computed tomography scanning at 2, 4 and 6 months of age does not induce damage to tibial bone microstructure in male and female CD-1 mice. BoneKEy reports. 2017;6:855–. 10.1038/bonekey.2016.87 . 28277563 PMC5234264

[14] Roberts BC, Giorgi M, Oliviero S, Wang N, Boudiffa M, Dall'Ara E. The longitudinal effects of ovariectomy on the morphometric, densitometric and mechanical properties in the murine tibia: A comparison between two mouse strains. Bone. 2019;127:260–70. 10.1016/j.bone.2019.06.024 31254730

[15] Kazakia GJ, Burghardt AJ, Cheung S, Majumdar S. Assessment of bone tissue mineralization by conventional x-ray microcomputed tomography: comparison with synchrotron radiation microcomputed tomography and ash measurements. MED PHYS. 2008;35(7):3170–9. 10.1118/1.2924210 18697542 PMC2673562

[16] Lu Y, Boudiffa M, Dall'Ara E, Bellantuono I, Viceconti M. Development of a protocol to quantify local bone adaptation over space and time: Quantification of reproducibility. Journal of Biomechanics. 2016;(49):2095–9. 27262181 10.1016/j.jbiomech.2016.05.022

[17] Oliviero S, Giorgi M, Dall'Ara E. Validation of finite element models of the mouse tibia using digital volume correlation. Journal of the Mechanical Behavior of Biomedical Materials. 2018;86:172–84. 10.1016/j.jmbbm.2018.06.022 29986291

[18] Birkhold AI, Razi H, Duda GN, Weinkamer R, Checa S, Willie BM. The influence of age on adaptive bone formation and bone resorption. Biomaterials. 2014;35(34):9290–301. 10.1016/j.biomaterials.2014.07.051 25128376

[19] Meijering EHW. Spline interpolation in medical imaging: comparison with other convolution-based approaches. 10th European Signal Processing Conference, Tampere. 2000:1–8. http://ieeexplore.ieee.org/stamp/stamp.jsp?tp=&arnumber=7075214&isnumber=7065137.

[20] Chen Y, Dall׳Ara E, Sales E, Manda K, Wallace R, Pankaj P, et al Micro-CT based finite element models of cancellous bone predict accurately displacement once the boundary condition is well replicated: A validation study. Journal of the Mechanical Behavior of Biomedical Materials. 2017;65:644–51. 10.1016/j.jmbbm.2016.09.014 27741494

[21] Christiansen BA. Effect of micro-computed tomography voxel size and segmentation method on trabecular bone microstructure measures in mice. Bone Reports. 2016;5:136–40. 10.1016/j.bonr.2016.05.006 27430011 PMC4926804

[22] Webster DJ, Morley PL, van Lenthe GH, Müller R. A novel in vivo mouse model for mechanically stimulated bone adaptation–a combined experimental and computational validation study. Computer Methods in Biomechanics and Biomedical Engineering. 2008;11(5):435–41. 10.1080/10255840802078014 18612871

[23] Pistoia W, van Rietbergen B, Lochmüller EM, Lill CA, Eckstein F, Rüegsegger P. Estimation of distal radius failure load with micro-finite element analysis models based on three-dimensional peripheral quantitative computed tomography images. Bone. 2002;30(6):842–8. 10.1016/s8756-3282(02)00736-6 12052451

[24] Bayraktar HH, Morgan EF, Niebur GL, Morris GE, Wong EK, Keaveny TM. Comparison of the elastic and yield properties of human femoral trabecular and cortical bone tissue. Journal of Biomechanics. 2004;37(1):27–35. 10.1016/s0021-9290(03)00257-4 14672565

[25] Judex S, Chung H, Torab A, Xie L, Rubin C, Donahue LR, et al., editors. Micro-CT induced radiation does not exacerbate disuse related bone loss2005 20052005.

[26] Margolis DS, Lien Y-HH, Lai L-W, Szivek JA. Bilateral symmetry of biomechanical properties in mouse femora. Medical Engineering & Physics. 2004;26(4):349–53. 10.1016/j.medengphy.2003.11.002. 15121061

[27] Main RP, Lynch ME, van der Meulen MCH. Load-induced changes in bone stiffness and cancellous and cortical bone mass following tibial compression diminish with age in female mice. The Journal of Experimental Biology. 2014;217(Pt 10):1775–83. 10.1242/jeb.085522 24577445 PMC4020944

