# Supplementary material for: Effect of repeated in vivo microCT imaging on the properties of the mouse tibia
Source: PLoS One. 2019 Nov 21;14(11):e0225127. doi: 10.1371/journal.pone.0225127 (PMC6874075; doi:10.1371/journal.pone.0225127)
Supplement: S1 Table — (DOCX) [file pone.0225127.s001.docx]

**S1 Table. Differences in local TMD between right (irradiated) and left (non-irradiated) tibiae.** B6 = C57BL/6 mice, BAL = BALB/c mice, WT = wild type, OVX = ovariectomy. Percentage differences (median ± SD) are reported for the ten longitudinal sections (01 = most proximal, 10 = most distal) and four quadrants (L = lateral, A = anterior, M = medial, P = posterior).

| **B6-WT** | |  |  |  |  | **B6-OVX** | |  |  |  |
| --- | --- | --- | --- | --- | --- | --- | --- | --- | --- | --- |
|  | **L** | **A** | **M** | **P** |  |  | **L** | **A** | **M** | **P** |
| **01** | -1±2 | 0±1 | 0±1 | -1±4 |  | **01** | -3±2 | 1±4 | -4±3 | -4±2 |
| **02** | 0±2 | 0±1 | 1±2 | 0±2 |  | **02** | -2±3 | -2±3 | -1±3 | 0±2 |
| **03** | 1±3 | 1±2 | 1±3 | -1±1 |  | **03** | -1±4 | 0±4 | 0±2 | 0±2 |
| **04** | 0±2 | 1±2 | 0±3 | -1±1 |  | **04** | -2±3 | 0±3 | 0±2 | 0±1 |
| **05** | 2±1 | 0±1 | -1±2 | -1±1 |  | **05** | 0±3 | 0±3 | -3±2 | -3±1 |
| **06** | 1±1 | 1±2 | 0±1 | 0±1 |  | **06** | 2±2 | 0±2 | -1±2 | 0±2 |
| **07** | 1±2 | 0±2 | 0±1 | 0±1 |  | **07** | 0±2 | 0±2 | 0±1 | 1±2 |
| **08** | 2±2 | -1±1 | 0±1 | 0±2 |  | **08** | 1±2 | 1±1 | -1±1 | 1±1 |
| **09** | 1±2 | 1±2 | 0±2 | 1±1 |  | **09** | 0±2 | 1±1 | -2±1 | 2±1 |
| **10** | 2±3 | 0±3 | 0±3 | -1±3 |  | **10** | 0±2 | 2±3 | -1±1 | 0±3 |
| **BAL-WT** | |  |  |  |  | **BAL-OVX** | |  |  |  |
|  | **L** | **A** | **M** | **P** |  |  | **L** | **A** | **M** | **P** |
| **01** | -1±2 | 0±2 | -2±3 | -1±2 |  | **01** | -3±2 | 0±3 | -1±2 | -1±1 |
| **02** | 0±2 | 0±0 | -1±4 | 0±1 |  | **02** | -1±1 | 1±1 | 1±4 | 0±2 |
| **03** | -2±2 | 1±1 | -2±3 | 0±1 |  | **03** | 0±1 | 1±1 | -1±3 | 0±1 |
| **04** | -1±3 | 1±1 | -2±3 | 0±1 |  | **04** | -1±1 | 1±1 | -1±2 | 1±1 |
| **05** | 0±3 | 0±1 | -1±2 | 0±1 |  | **05** | -2±2 | -1±2 | -1±2 | 0±1 |
| **06** | 0±2 | 0±2 | 1±2 | 0±2 |  | **06** | -1±1 | 2±3 | 0±1 | 0±1 |
| **07** | 0±2 | 0±1 | 1±1 | 1±2 |  | **07** | 0±1 | 1±2 | 0±2 | 1±1 |
| **08** | 0±2 | 0±1 | 0±1 | 1±2 |  | **08** | -1±0 | 1±1 | -1±1 | 0±1 |
| **09** | 1±2 | 2±2 | 0±1 | 1±2 |  | **09** | 1±1 | 2±1 | 0±1 | 0±1 |
| **10** | 0±3 | 1±3 | 0±1 | -1±1 |  | **10** | 0±1 | 2±3 | -1±2 | 0±1 |
