# Supplementary material for: Effect of repeated in vivo microCT imaging on the properties of the mouse tibia
Source: PLoS One. 2019 Nov 21;14(11):e0225127. doi: 10.1371/journal.pone.0225127 (PMC6874075; doi:10.1371/journal.pone.0225127)
Supplement: S2 Table — (DOCX) [file pone.0225127.s002.docx]

**S2 Table. Differences in local BMD between right (irradiated) and left (non-irradiated) tibiae.** B6 = C57BL/6 mice, BAL = BALB/c mice, WT = wild type, OVX = ovariectomy. Percentage differences (median ± SD) are reported for the ten longitudinal sections (01 = most proximal, 10 = most distal) and four quadrants (L = lateral, A = anterior, M = medial, P = posterior).

| **B6-WT** | |  |  |  |  | **B6-OVX** | |  |  |  |
| --- | --- | --- | --- | --- | --- | --- | --- | --- | --- | --- |
|  | **L** | **A** | **M** | **P** |  |  | **L** | **A** | **M** | **P** |
| **01** | 1±7 | 1±8 | 4±9 | -5±2 |  | **01** | -7±9 | -6±11 | -4±8 | -10±5 |
| **02** | 2±8 | -2±6 | 5±7 | 1±19 |  | **02** | -2±10 | -4±7 | -4±6 | -1±5 |
| **03** | 1±5 | -1±6 | -1±6 | 2±21 |  | **03** | -2±11 | -3±7 | -2±9 | 2±7 |
| **04** | -2±5 | -2±8 | -2±11 | 1±22 |  | **04** | -9±11 | -5±7 | -9±12 | 1±7 |
| **05** | 2±5 | -3±6 | 2±7 | 5±19 |  | **05** | -3±9 | 0±5 | -3±8 | 1±12 |
| **06** | 1±3 | 1±5 | 0±3 | 0±5 |  | **06** | 4±5 | 2±4 | -2±6 | -2±3 |
| **07** | 1±8 | 3±5 | 2±3 | 0±4 |  | **07** | 5±4 | 2±10 | 0±4 | 1±7 |
| **08** | 3±4 | 3±1 | 2±4 | 0±5 |  | **08** | -2±6 | 1±5 | -5±5 | 0±10 |
| **09** | -1±5 | 1±3 | -2±3 | 2±3 |  | **09** | -3±7 | 1±4 | -9±6 | 0±5 |
| **10** | 5±6 | 3±8 | 1±3 | 0±2 |  | **10** | -3±8 | -1±10 | -3±5 | -4±5 |
| **BAL-WT** | |  |  |  |  | **BAL-OVX** | |  |  |  |
|  | **L** | **A** | **M** | **P** |  |  | **L** | **A** | **M** | **P** |
| **01** | -5±7 | -3±11 | 6±14 | 7±9 |  | **01** | -4±8 | 0±8 | -8±9 | 1±7 |
| **02** | -3±8 | 2±4 | 2±9 | 5±5 |  | **02** | 6±10 | 7±4 | 0±4 | 4±4 |
| **03** | -2±3 | 4±3 | 1±4 | 0±3 |  | **03** | -1±3 | 4±2 | 4±2 | 5±2 |
| **04** | -4±5 | 7±4 | 1±4 | 1±4 |  | **04** | -3±1 | 6±1 | 4±2 | 6±4 |
| **05** | 0±4 | 5±6 | 4±5 | 2±2 |  | **05** | -2±4 | 3±0 | 5±4 | 2±3 |
| **06** | -3±2 | 0±2 | 3±2 | -1±2 |  | **06** | -2±4 | 3±3 | 3±3 | 2±2 |
| **07** | -3±3 | 1±2 | 3±2 | -1±2 |  | **07** | -1±3 | 2±4 | 1±4 | -2±4 |
| **08** | 0±4 | 2±2 | 2±1 | 0±2 |  | **08** | 3±2 | 0±3 | 2±3 | 5±2 |
| **09** | 2±5 | 2±4 | 4±2 | 2±4 |  | **09** | 4±2 | 4±3 | 2±4 | 2±3 |
| **10** | 1±4 | 1±1 | 5±5 | 4±3 |  | **10** | -2±4 | 1±8 | 6±5 | 2±4 |
