# Supplementary material for: Effect of repeated in vivo microCT imaging on the properties of the mouse tibia
Source: PLoS One. 2019 Nov 21;14(11):e0225127. doi: 10.1371/journal.pone.0225127 (PMC6874075; doi:10.1371/journal.pone.0225127)
Supplement: S3 Table — (DOCX) [file pone.0225127.s003.docx]

**S3 Table. Differences in local BV/TV between right (irradiated) and left (non-irradiated) tibiae.** B6 = C57BL/6 mice, BAL = BALB/c mice, WT = wild type, OVX = ovariectomy. Percentage differences (median ± SD) are reported for the ten longitudinal sections (01 = most proximal, 10 = most distal) and four quadrants (L = lateral, A = anterior, M = medial, P = posterior).

| **B6-WT** | |  |  |  |  | **B6-OVX** | |  |  |  |
| --- | --- | --- | --- | --- | --- | --- | --- | --- | --- | --- |
|  | **L** | **A** | **M** | **P** |  |  | **L** | **A** | **M** | **P** |
| **01** | 1±8 | 1±8 | 4±9 | -3±4 |  | **01** | -5±7 | -2±10 | -1±5 | -9±4 |
| **02** | 1±7 | -1±6 | 4±7 | -1±22 |  | **02** | -5±8 | -3±6 | -7±7 | -2±4 |
| **03** | 0±6 | -1±5 | -2±7 | 2±22 |  | **03** | -3±9 | -3±5 | -2±8 | 1±6 |
| **04** | 0±5 | -4±7 | -1±12 | 1±22 |  | **04** | -7±8 | -5±5 | -8±10 | 0±6 |
| **05** | 0±4 | -3±6 | 3±8 | 6±20 |  | **05** | -2±6 | 0±3 | 0±6 | 3±12 |
| **06** | -1±2 | -1±4 | 1±4 | 1±6 |  | **06** | 2±3 | 2±2 | -1±4 | 0±2 |
| **07** | 0±7 | 2±5 | 1±3 | 0±4 |  | **07** | 4±4 | 3±9 | 0±3 | 1±8 |
| **08** | 1±3 | 4±2 | 2±4 | 0±4 |  | **08** | -2±4 | 0±4 | -2±4 | -2±9 |
| **09** | -3±4 | -1±3 | -2±2 | 1±2 |  | **09** | -2±6 | 0±4 | -6±5 | -2±4 |
| **10** | 2±4 | 1±7 | 1±1 | 1±2 |  | **10** | -4±7 | -3±8 | -2±4 | -2±4 |
| **BAL-WT** | |  |  |  |  | **BAL-OVX** | |  |  |  |
|  | **L** | **A** | **M** | **P** |  |  | **L** | **A** | **M** | **P** |
| **01** | -6±8 | -3±9 | 6±17 | 8±10 |  | **01** | -2±9 | -1±7 | -9±10 | 3±7 |
| **02** | -5±9 | 2±4 | 4±12 | 4±5 |  | **02** | 7±8 | 6±3 | -2±6 | 4±4 |
| **03** | -3±3 | 3±3 | 1±5 | 0±4 |  | **03** | -2±3 | 3±1 | 3±3 | 5±1 |
| **04** | -3±3 | 5±4 | 3±4 | 0±4 |  | **04** | -3±2 | 6±0 | 3±3 | 4±4 |
| **05** | -1±3 | 5±6 | 5±4 | 3±3 |  | **05** | -3±4 | 4±1 | 5±2 | 3±3 |
| **06** | -3±2 | 0±3 | 2±2 | -1±3 |  | **06** | -1±3 | 0±2 | 3±3 | 2±2 |
| **07** | -4±2 | 1±2 | 3±2 | -1±2 |  | **07** | -2±3 | 2±4 | 1±3 | -3±5 |
| **08** | -1±2 | 1±2 | 2±1 | 1±2 |  | **08** | 3±2 | 0±2 | 1±3 | 4±3 |
| **09** | 1±3 | 4±3 | 3±2 | 1±2 |  | **09** | 2±2 | 2±2 | 2±3 | 1±2 |
| **10** | 2±2 | 1±3 | 3±5 | 3±2 |  | **10** | -1±5 | -2±7 | 7±3 | 2±4 |
