# Supplementary material for: Effect of repeated in vivo microCT imaging on the properties of the mouse tibia
Source: PLoS One. 2019 Nov 21;14(11):e0225127. doi: 10.1371/journal.pone.0225127 (PMC6874075; doi:10.1371/journal.pone.0225127)
Supplement: S1 Appendix — (DOCX) [file pone.0225127.s004.docx]

**S1 Appendix. Nominal radiation dose**

The nominal radiation dose associated with the scanning procedure applied in this study has been calculated using data provided by the manufacturer of the scanner. The radiation dose associated with a reference scan *(y)* was provided, characterised by the same energy and current. Since radiation dose is linearly proportional to integration time (IT) and number of projections, the nominal radiation dose associated with the scanning procedure applied in this study was calculated with a simple proportion equation:

$$Radiation dose\left( z \right)= \frac{Radiation dose\left( y \right) IT\left( z \right) Projections(z)}{IT\left( y \right) Projections(y)}$$
